# Reconstruction of the anatomy of the hip in total hip arthroplasty with two different kinds of stems

**DOI:** 10.1186/s12891-022-05152-9

**Published:** 2022-03-05

**Authors:** Bernd Fink, Mohamed Morgan, Philipp Schuster

**Affiliations:** 1 Department for Joint Replacement, Rheumatoid and General Orthopaedics, Orthopaedic Clinic Markgröningen, Kurt-Lindemann-Weg 10, 71706 Markgröningen, Germany; 2 Orthopaedic Department, University Hospital Hamburg-Eppendorf, Martinistrasse 52, 20246 Hamburg, Germany; 3 Department of Orthopedics and Traumatology, Paracelsus Medical Private University, Clinic Nuremberg, Nuremberg, Germany

**Keywords:** Total hip replacement, Prosthetic stem, Anatomy

## Abstract

**Background:**

The reconstruction of the individual anatomy is important in total hip replacement. The aim of the study was to compare two different kinds of stems with respect to the reconstruction of the individual anatomy of the hip.

**Methods:**

We compared the restoration of the anatomical parameters (horizontal and vertical offset, femoral neck-shaft angle (NSA) and leg length) of 100 unilateral CoreHip (CH) implantations with 100 unilateral implantations of a standard anatomical stem (Exception (E)). The CoreHip has three different NSAs and exhibits a constant femoral neck length for the different sizes. The Exception stem has a standard and lateralized version with two different NSAs and, in both versions, the femoral neck length increases proportionately with size. The anatomical parameters of the operated and healthy sides were measured and the differences between the two stems compared.

**Results:**

The horizontal (2.5 ± 2.8 mm (mean ± SD) for CH vs. 5.4 ± 4.1 mm for E, *p* < 0.001) and vertical offset (4.1 ± 3.5 mm for CH vs. 5.0 ± 3.8 mm, *p* = 0.024) and femoral neck-shaft-angle (1.7 ± 1.6 degrees for CH vs. 5.6 ± 3.4 degrees for E, *p* < 0.001) could be reconstructed significantly better with the CoreHip system. There was a tendency for the leg length (4.0 ± 3.9 mm for CH vs. 4.5 ± 3.8 mm; *p* = 0.11) to be better restored with the CoreHip.

**Conclusion:**

The reconstruction of the individual anatomy of the hip with an endoprosthesis could be realized significantly better with the stem that was designed with three different femoral neck-shaft angles and a constant femoral neck length over different sizes.

## Background

Artificial hip joint replacement is one of the most successful and common surgical procedures in medicine and has been called “the operation of the century” [1]. Correct reconstruction of the individual anatomy with respect to femoral neck shaft angle, horizontal and vertical offset with the endoprosthesis are important goals of this procedure [2–5].

Anatomical studies have shown that the distribution of the femoral neck-shaft-angle follows a Gaussian distribution, with a mean of approximately 126 degrees (i.e. significantly lower than the 135 degrees used in most hip prosthesis stems) and physiological values between 108 and 145 degrees [6–9]. There are also differences between various ethnic groups, with higher mean values for the African (about 131 degrees) and Asian regions (about 134 degrees) [8, 10–12]. Furthermore, the length of the femoral neck plays a role, and changes in its length have an influence on both leg length and offset [7].

Most prosthesis stems available on the market have two ranges with different femoral neck-shaft angles (NSA), a standard version (NSA usually 135 degrees) and a more lateral version (NSA usually about 126 to 128 degrees) with higher offset. In both versions the neck length increases with the stem size, i.e. with the stem diameter, which in turn increases the offset and leg length. In nature, however, this linear relationship between femoral neck length and stem diameter (or size) does not exist, so that the same offsets and femoral neck lengths can exist for very different widths of the femoral canal, and vice versa [13–16]. In addition, the width of the femoral canal may change over the course of a lifetime. Thus, especially in women with progressive osteoporosis, the femoral canal becomes wider, but the other anatomical parameters such as offset, femoral neck length and leg length remain the same [17]. The continual increase in the length of the femoral neck with increasing canal width in the vast majority of prosthetic stems on the market doesn’t take this phenomenon into account.

Therefore, the current study was designed to test the hypothesis, that a prosthetic stem with three different femoral neck-shaft angles and a constant length of the femoral neck in different sizes can restore the individual anatomy of the femoral joint (horizontal and vertical femoral offset as well as neck-shaft-angle) significantly better than a standard prosthetic stem where femoral neck length increases with size and with two different NSAs (standard and lateralized).

## Methods

**Fig. 1:**
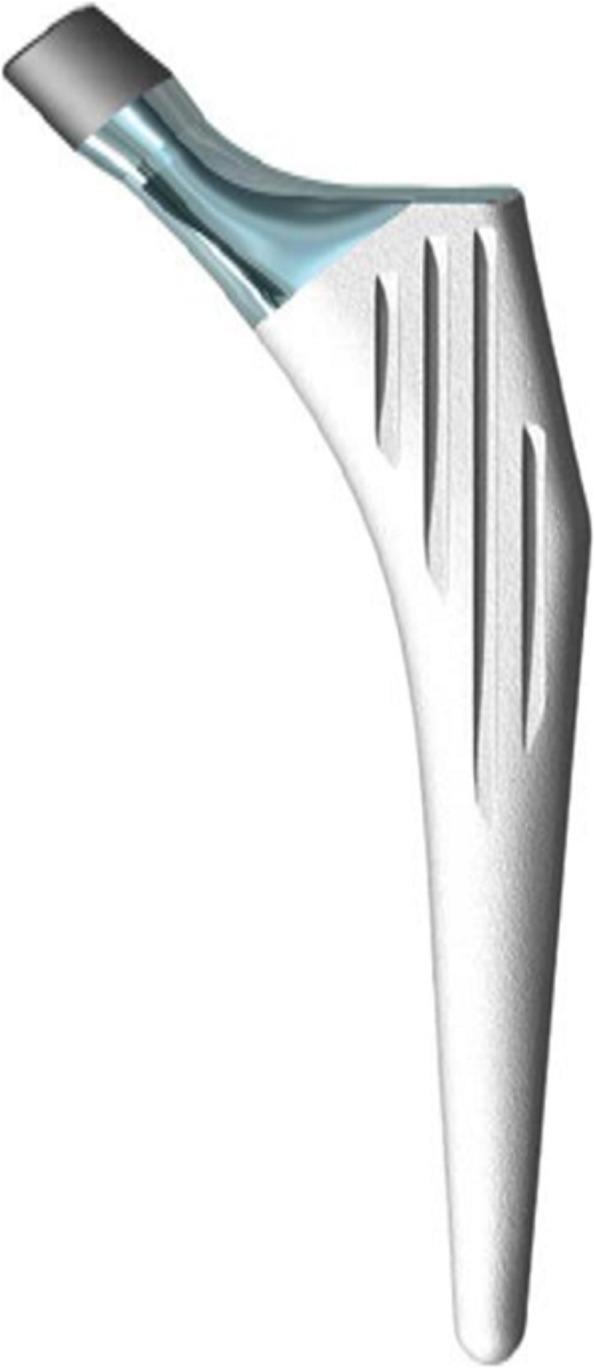
Exception stem

For the standard version of a prosthetic stem the anatomical stem Exception (ZimmerBiomet, Winterthur, Switzerland) with a right and left version was chosen. This prosthetic stem exhibit a femoral neck-shaft-angle of 137.5 degrees in its standard version whereas, in the lateralized version, the femoral neck-shaft angle changed with the size of the stem from 125.4 degrees to 130 degrees (Fig. 1).

**Fig. 2:**
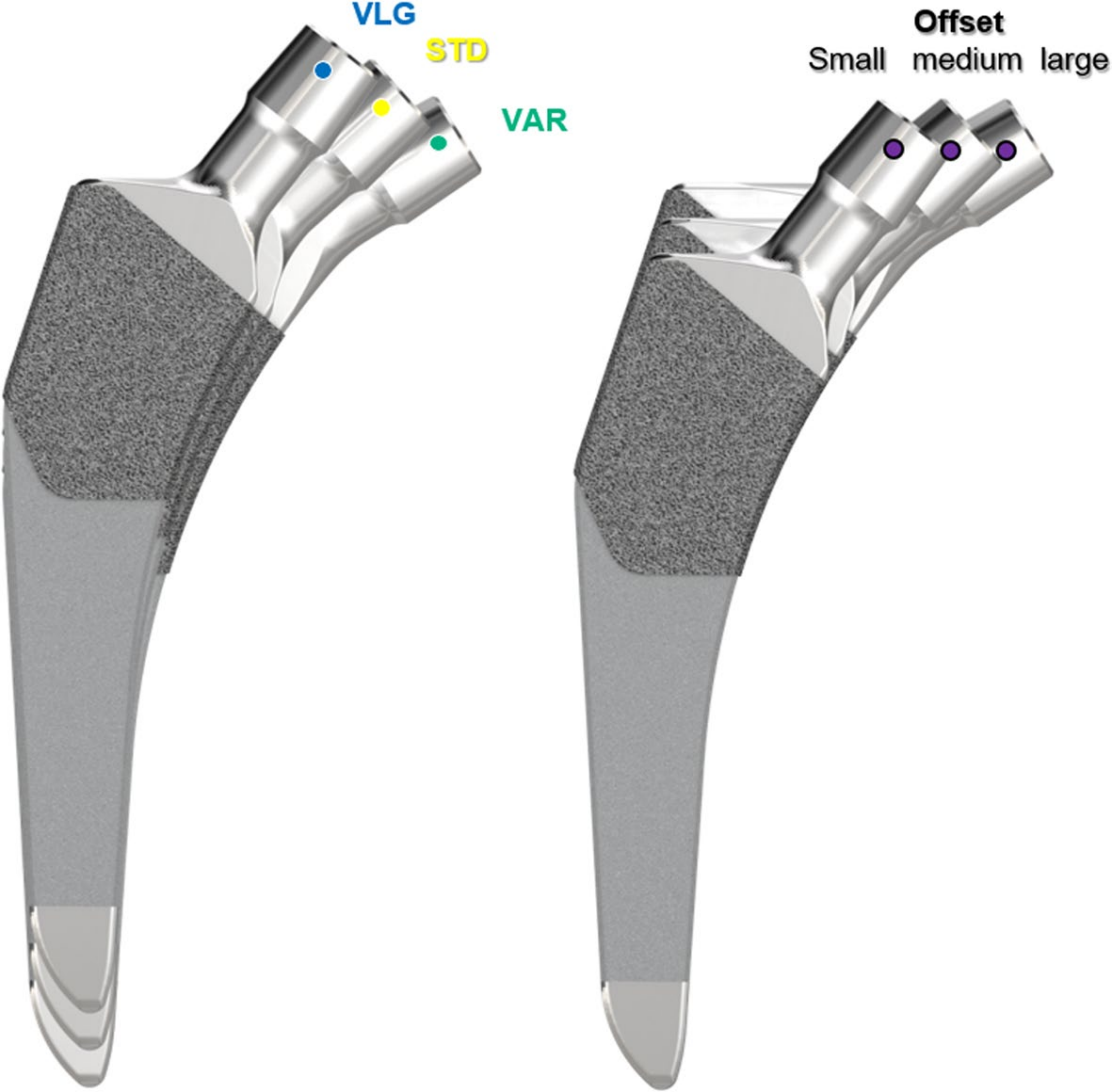
Setting of three different offsets for the same stem size and leg length in the CoreHip-System

The other stem selected was the CoreHip (Aesculap, Tuttlingen, Germany). The CoreHip system is a prosthetic stem system in which each size has three different femoral neck-shaft angles (Varus 122°, Standard 132°, Valgus 142°) (Fig. 2). These can all be implanted with one rasp of the corresponding size. In this system the neck length does not increase with increasing stem size. If needed, it is also possible to achieve a greater femoral neck length with the XL head variant of this system (also available in ceramic).

The cementless Allofit classic cup (ZimmerBiomet, Winterthur, Switzerland) was used in both groups.

We compared the restoration of the anatomical parameters of 100 unilateral CoreHip implantations with 100 unilateral implantations of the standard anatomical stem (Exception). The patients were randomized to one of the two stems. There were 57 woman and 43 men in the CoreHip-group with an age of 65.5 ± 7.3 years and 60 woman and 40 men in the Exception-group with an age of 67.2 ± 7.9 years. Although no group size calculation has been performed beforehand a number of 100 cases per group were chosen as this represents the number of other similar studies in the literature and patients could be included within a manageable period of time of several months [18–20].

After assigning the patient to the specific stem design, preoperative planning was performed with the mediCAD 2D Classic System (mediCAD Hectec GmbH, Altdorf/Landshut, Germany) for the corresponding implants to be used. In addition to the size selection and positioning of the respective implant, a preselection was made for the stem variant to be used (standard or varus for the Exception stem and standard, varus or valgus for the CoreHip stem). This was verified during intraoperative testing with the trial implants. The operations with both stems were performed by four highly experienced surgeons.

**Fig. 3:**
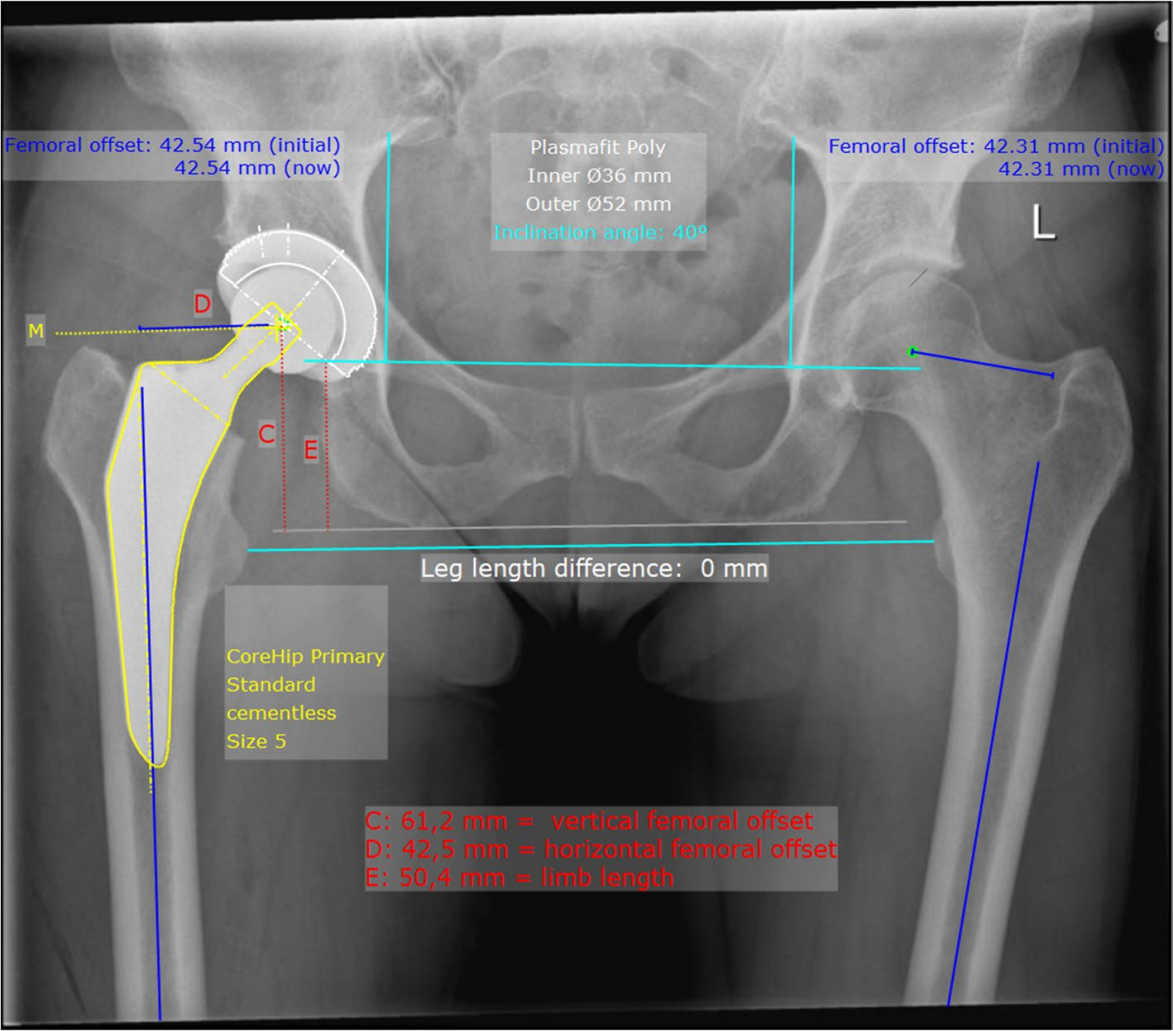
Anatomical parameters, for the comparison of hip prosthesis implantation with the contralateral, non-operated side

**Fig. 4:**
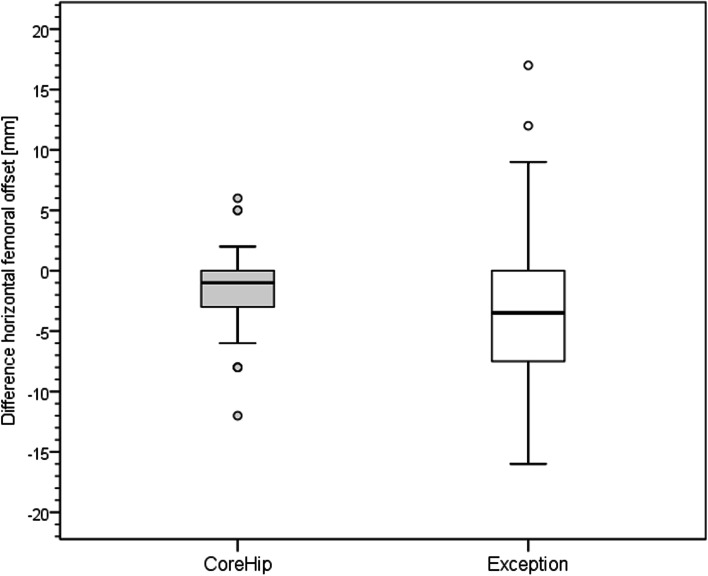
Distribution of the horizontal offset of the CoreHip and Exception prosthesis. Y-axis = difference betweenhealthy and operated side (healthy - operated) in mm

**Fig. 5:**
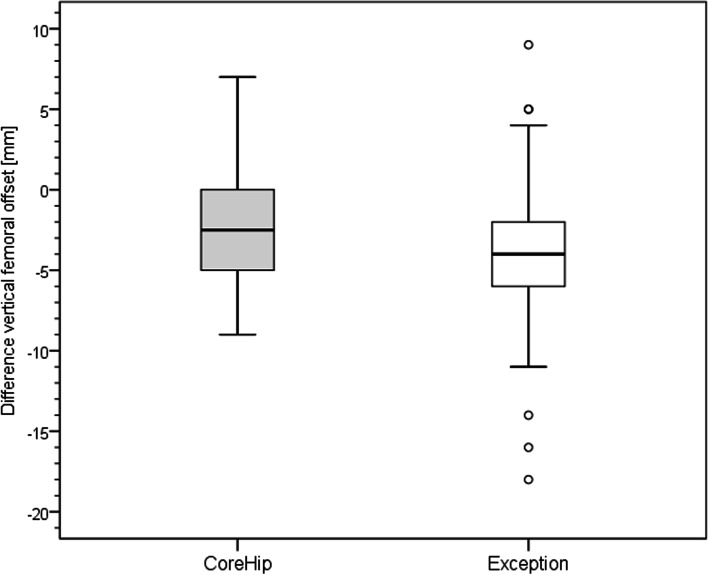
Distribution of the vertical offset of the CoreHip and Exception prosthesis. Y-axis = difference between healthy and operated side (healthy - operated) in mm

**Fig. 6:**
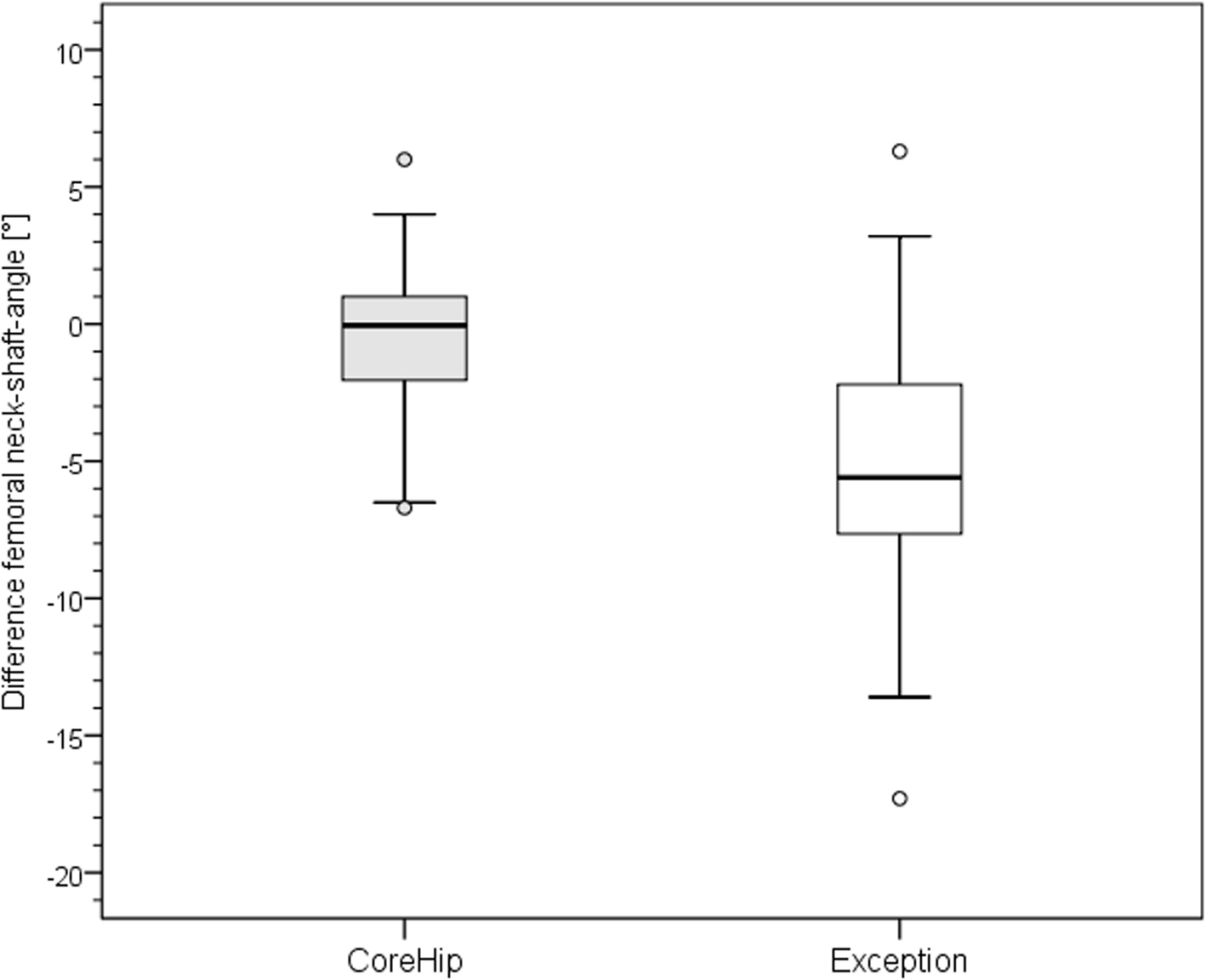
Distribution of the femoral neck-shaft angle (NSA) of the CoreHip and Exception prosthesis. Y-axis = difference between healthy and operated side (healthy - operated) in degrees

**Fig. 7:**
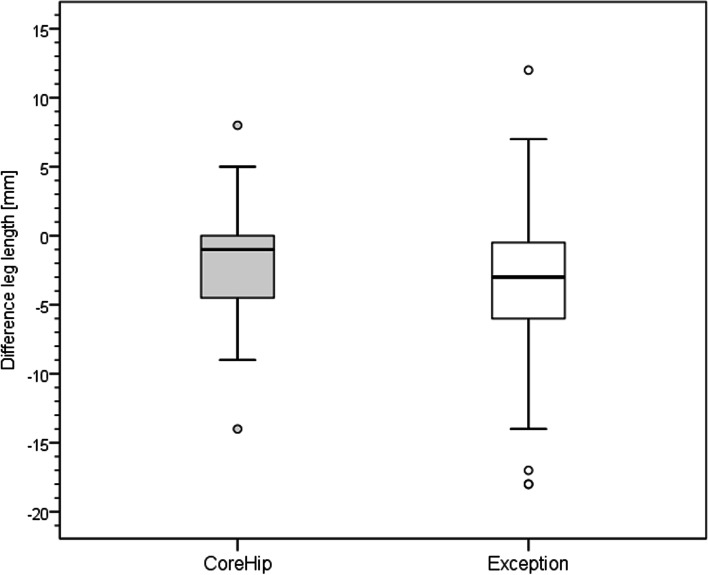
Distribution of the leg length with the CoreHip and Exception prosthesis. y-axis = difference between healthy and operated side (healthy - operated) in mm

On the postoperative radiographic pelvic overview (performed 5 days after surgery) with a film-focus-distance of 115 cm the following parameters were measured on the replaced hip and on the healthy contralateral side (Fig. 3): femoral neck-shaft angle, vertical offset (C), horizontal offset (D) and leg length (E). The prosthetic head diameter was used for correction of the magnification. The difference between these anatomical parameters on the healthy side and the operated side was calculated for each patient and taken as real values for the graphical representation of the distribution (Figs. 4, 5, 6 and 7) and as integers for statistical analysis (Table 2). Here, the value describes the extent of deviation of the operated side from the healthy side.

Further, the accuracy of restoring anatomy within defined limits (5 mm for vertical and horizontal offset, 3° for femoral neck-shaft-angle) was compared between the two groups and the fraction calculated in each group that could be reconstructed within those limits.

### Statistical analysis

Statistical evaluation was performed using SPSS for Windows (version 22; IBM Corp.; Armonk, NY). For statistical evaluation of nonparametric data of unrelated samples the Mann-Whitney-U-Test was used. The chi-square test and Fisher’s exact test were used for statistical evaluation of categorial variables. All reported *p*-values are two-tailed, with an alpha level < 0.05 considered significant.

## Results

There was no difference between the two stem groups when the anatomical parameters horizontal and vertical femoral offset, as well as the neck-shaft-angle (NSA) on the healthy side were compared (Fig. 3, Table 1). On the operated side, it was found that the horizontal and vertical offset as well as the femoral neck-shaft-angle (NSA) could be reconstructed significantly better with the CoreHip system and also that there was a tendency for the leg length to be better restored with the CoreHip (Figs. 3, 4, 5, 6 and 7, Table 2). It also became apparent that the horizontal offset could be reconstructed within a limit of 5 mm for the CoreHip in 91% of cases, but only in 63% of cases for the anatomical stem within the same limit (*p* < 0.001, chi-square test). The femoral neck-shaft-angle could be reconstructed within 3 degrees with the CoreHip in 92% of the cases, but only in 43% of the cases for the anatomical stem (*p* < 0.001, chi-square test). The vertical offset could be reconstructed within 5 mm with the CoreHip in 70% of cases and with the anatomical stem in 64% of cases (*p* = 0.366, chi-square test). The leg length was within 5 mm for the CoreHip in 80% of cases and 69% of cases for the anatomical stem (*p* = 0.074, chi-square test).

**Table 1:** Comparison of the anatomical parameters on the healthy hip of both groups

	CoreHIP Healthy side	Anatomical stem Healthy side	***p***-value (Mann-Whitney-U-Test)
offset horizontal	42 ± 8 mm (23 – 63 mm)	43 ± 7 mm (25 – 60) mm	*p* = 0.731
femoral neck-shaft-angle	131 ± 6 degrees (118 – 143 degrees)	130 ± 5 degrees (118 – 141 degrees)	*p* = 0.428
offset vertical	63 ± 6 mm (53 – 79 mm)	62 ± 7 mm (27 – 80 mm)	*p* = 0.667
LLD	48 ± 7 mm (32 – 67 mm)	49 ± 6 mm (34 – 65 mm)	*p* = 0.382

*LLD* leg length discrepancy). Mean ± standard deviation (minimum – maximum) is shown

**Table 2:** Comparison of the restoration of anatomical parameters between the CoreHip stem and a standard anatomical stem (Δ = difference between the contralateral, non-operated side and the same parameter on the operated side after total hip arthroplasty, LLD = leg length discrepancy). Mean ± standard deviation (minimum – maximum) is shown

	CoreHIP	Anatomical stem	***p***-value (Mann-Whitney-U-Test)
Δ offset horizontal	2.5 ± 2.8 mm (0 – 14 mm)	5.4 ± 4.1 mm (0 – 17 mm)	*p* < 0.001
Δ femoral neck-shaft-angle	1.7 ± 1.6 degrees (0 – 6.7 degrees)	5.6 ± 3.4 degrees (0.2 – 17.1 degrees)	*p* < 0.001
Δ offset vertical	4.1 ± 3.5 mm (0 - 15 mm)	5.0 ± 3.8 mm (0 – 25 mm)	*p* = 0.024
Δ LLD	4.0 ± 3.9 mm (0 - 15 mm)	4.5 ± 3.8 mm (0 – 18 mm)	*p* = 0.10

## Discussion

The comparison of the two different types of prosthetic stems showed, that the stem with three different femoral neck-shaft angles and a constant femoral neck length could reconstruct the individual anatomical parameters significantly better for the horizontal and vertical offset as well as for the femoral neck-shaft angle and tendentially better for the leg length.

The better reconstruction of the anatomical parameters by the CoreHip stem is in our view founded on two characteristics of this stem system. First, by using three different CCD angle variants of the stem, that have the same medial calcar fit, the corresponding anatomical offset can be reconstructed without affecting the leg length (Fig. 2). Second, with the CoreHip system, the prosthetic neck length does not increase with increasing stem thickness, as is the case with many other stem systems on the market (including the Exception stem studied here). This corresponds to the pattern found in nature, since in nature there is no linear relationship between femoral neck length and the femoral canal width [13–16]. Moreover, especially in women with progressive osteoporosis, the femoral canal can become wider over the course of a lifetime, but the other anatomical parameters such as offset, femoral neck length and leg length remain the same [17]. Especially in the latter condition, the use of a stem-system with increasing neck lengths with stem sizes would result in a significant increase in the offset.

An increase in the offset can have clinical consequences. It leads to tightness of the iliotibial ligament, which in turn can lead to irritation of the gluteal muscles at the greater trochanter and result in bursitis trochanterica [21]. An increase in the horizontal femoral offset after hip prosthesis implantation can also lead to a change in the leg axis with a resulting change in the forces on the knee joint and even to unilateral osteoarthritis of the knee [22]. On the other hand, a reduction of the offset reduces the tension of the gluteal muscles, which can lead to a Trendelenburg sign or gait [23]. In addition, the distance of the trochanter major to the os ileum and that of the trochanter minor to the os ischium is reduced, and this can lead to bony impingement or even result in dislocation of the hip joint [24]. Furthermore, a reduced offset increases the wear of polyethylene in the cup [25–27]. Therefore, it seems that the correct reconstruction of the offset has clear clinical advantages.

In addition to the offset, the leg length plays a crucial role in the reconstruction of the anatomical parameters. A changed leg length leads to pelvic tilt, which, if not compensated for by insoles or shoe adjustment, can cause problems in the lumbar spine and irritation of the N. ischiadicus [28–30]. In addition, differences in leg length may cause gait insecurity, dislocation and premature loosening of the prostheses after hip replacement [31–33]. Patient dissatisfaction with leg length discrepancies often leads to legal disputes [24–37]. Although the length of the prosthesis neck also has an influence on the leg length, the reconstruction of the leg length in this study was not significantly different between the two different stem systems. However, with the CoreHip, the leg length tended to be better reconstructed (11% more often) within a 5 mm difference to the non-operated opposite side. The lack of significance with regard to leg length, despite significant differences in the reconstruction of the vertical femoral offset, may in our opinion be due to the fact that the surgeons selected a larger or smaller stem to achieve the same leg length by placing the stem higher or lower in the femoral canal and could thus influence the leg length directly. The femoral offset was only slightly influenced by this. In addition, the use of modular prosthetic heads helps to achieve the correct insertion length.

From the explanations given, it seems evident that the reconstruction of the individual anatomy of the hip with the endoprosthesis is important and seems to be realized significantly better with a stem with three different femoral neck-shaft angles and a constant femoral neck length in different sizes. However, further studies should be aimed at determining whether the better reconstruction of the individual anatomical parameters also leads to different clinical outcomes.

The study has some limitations. First of all, this is a study of total endoprostheses. As such, the placement of the prosthetic cup also has an effect on leg length. Since only femoral vertical and horizontal offset was measured in this study and the same cup was used for both prosthetic stems, we believe that there is negligible influence of prosthetic cup placement on the significant differences detected in this study.

Moreover, the selected standard stem is not representative of all standard stems on the market because each stem has some specific features in neck-shaft angle and the increase in neck length with increasing stem size. However, it seems that an increase of neck-length with stem size generally does not reflect the natural anatomical relationship between the meta- and diaphyseal canal width and the femoral neck length [13–16].

## Conclusions

Therefore, a prosthetic stem with a constant prosthetic neck length and the possibility of three different NSAs seems to reconstruct the individual anatomical situation better. This conclusion should be considered during the development of prosthetic stems in the future.

## Data Availability

We do not wish to share our data, because some of patient’s data regarding individual privacy, and according to the policy of our hospital, the data could not be shared to others without permission.

## Abbreviation

NSA: Neck-shaft angle
